# Supplementary material for: Is Polymicrobial Bacteremia an Independent Risk Factor for Mortality in Acinetobacter baumannii Bacteremia?
Source: J Clin Med. 2020 Jan 6;9(1):153. doi: 10.3390/jcm9010153 (PMC7019703; doi:10.3390/jcm9010153)

Table. S1. Logistic regression analysis of predictors for 30-day mortality among patients with *Acinetobacter baumannii* bacteremia.

Demographic or characteristic	Univariable analysis		Multivariable analysis	
	**Odds ratio (95% CI)**	***p***	**Odds ratio (95% CI)**	***p***
Chronic kidney disease	1.412 (0.845–2.360)	0.188		
Coronary artery disease	1.853 (1.045–3.287)	0.035		
Malignancy	1.317 (0.848–2.045)	0.221	1.971 (1.083–3.588)	0.026
Neutropenia	3.870 (1.418–10.560)	0.008		
Recent surgery (within 4 weeks)	0.525 (0.328–0.840)	0.007		
Shock within 3 days	3.824 (1.279–11.432)	0.016		
APACHE II score	1.152 (1.117–1.189)	<0.001	1.156 (1.114–1.201)	<0.001
Arterial catheter	2.273 (1.364–3.788)	0.002		
Central venous catheter	2.079 (1.349–3.203)	0.001		
Foley catheter	2.606 (1.655–4.102)	<0.001		
Nasogastric tube	2.814 (1.709–4.633)	<0.001		
Ventilator	2.649 (1.710–4.101)	<0.001		
Respiratory tract infection	2.175 (1.400–3.379)	0.001		
Urinary tract infection	0.245 (0.084–0.717)	0.010		
Appropriate antimicrobial therapy	0.720 (0.455–1.138)	0.159	0.532 (.288–.983)	0.044
Polymicrobial bacteremia	0.864 (0.529–1.413)	0.561		

Abbreviations: ICU, intensive care unit; APACHE II, Acute Physiologic and Chronic Health Evaluation II; CI, confidence interval.

Table S2. Concomitantly isolated bacterial species and the appropriate antimicrobial regimens used for polymicrobial *Acinetobacter baumannii* bacteremia.

Concomitantly isolated bacterial species	Appropriate antimicrobial regimens
Coagulase-negative staphylococci	Imipenem + Vancomycin
Enterococcus spp.	Meropenem + Vancomycin
Enterococcus spp.	Meropenem + Teicoplanin
Staphylococcus aureus	Ceftazidime + Ciprofloxacin
Coagulase-negative staphylococci and *Enterococcus* spp.	Cefepime + Teicoplanin
Pseudomonas aeruginosa	Imipenem
Pseudomonas aeruginosa	Cefepime
Enterobacter spp.	Ceftazidime
Escherichia coli	Meropenem
Proteus mirabilis	Meropenem
Serratia marcescens	Ceftazidime
Yeast	Meropenem + Fluconazole

Table S3. Resistant rates of the concomitant pathogens in polymicrobial bacteremia.

Concomitant pathogen(s)	Case number (resistance rate)
MRSA	3 (75%)
MRSA + *Enterococcus spp.*	3 (50%)
MRSA + Coagulase-negative staphylococci	2 (100%)
ESBL-*K. pneumoniae*	3 (16.7%)
ESBL-*E. coli*	1 (14.3%)
AmpC-producing *E. coli*	1 (14.3%)
Metallo-beta-lactamases-producing organisms	0 (0%)

Figure S1. Kaplan–Meier plot showing the survival rates at 30 days of patients with acquired monomicrobial *Acinetobacter baumannii* (Ab) bacteremia, polymicrobial Ab bacteremia with concomitant Gram-negative bacilli (GNB) and polymicrobial Ab bacteremia with concomitant Gram-positive cocci (GPC) (polymicrobial Ab bacteremia with concomitant GNB versus monomicrobial Ab bacteremia, $p = 0.301$ by log-rank test; polymicrobial Ab bacteremia with concomitant GPC versus monomicrobial Ab bacteremia, $p = 0.545$ by log-rank test).

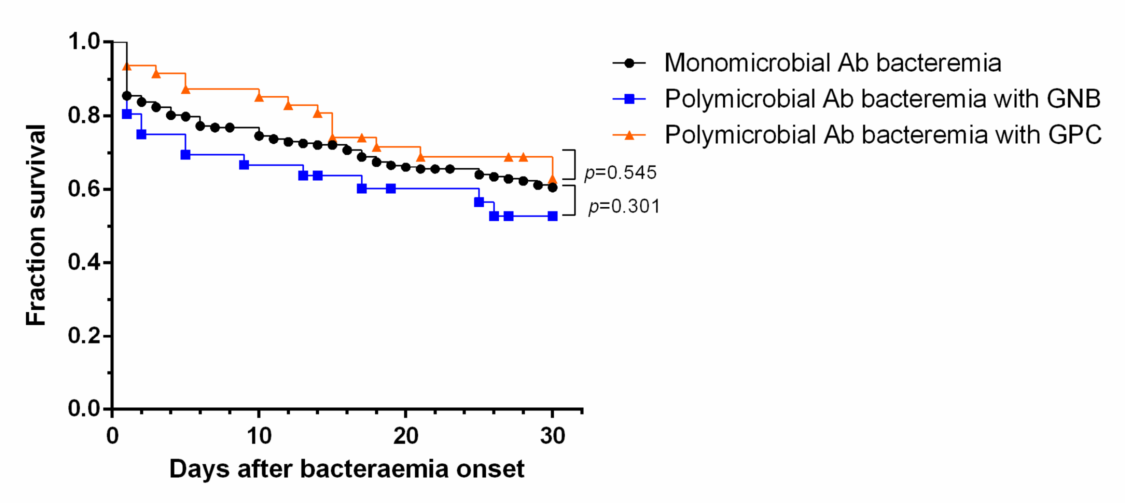

Figure S2. Kaplan–Meier plot showing the survival rates of patients with acquired polymicrobial multidrug-resistant *Acinetobacter baumannii* (MDRAb) bacteremia with concomitant Gram-negative bacilli (GNB), polymicrobial Ab bacteremia with concomitant Gram-positive cocci (GPC), and those with acquired monomicrobial MDRAb bacteremia. p-values were calculated by log-rank tests. (polymicrobial MDRAb bacteremia with concomitant GNB versus polymicrobial MDRAb bacteremia with concomitant GPC, p<0.001 by log-rank test).

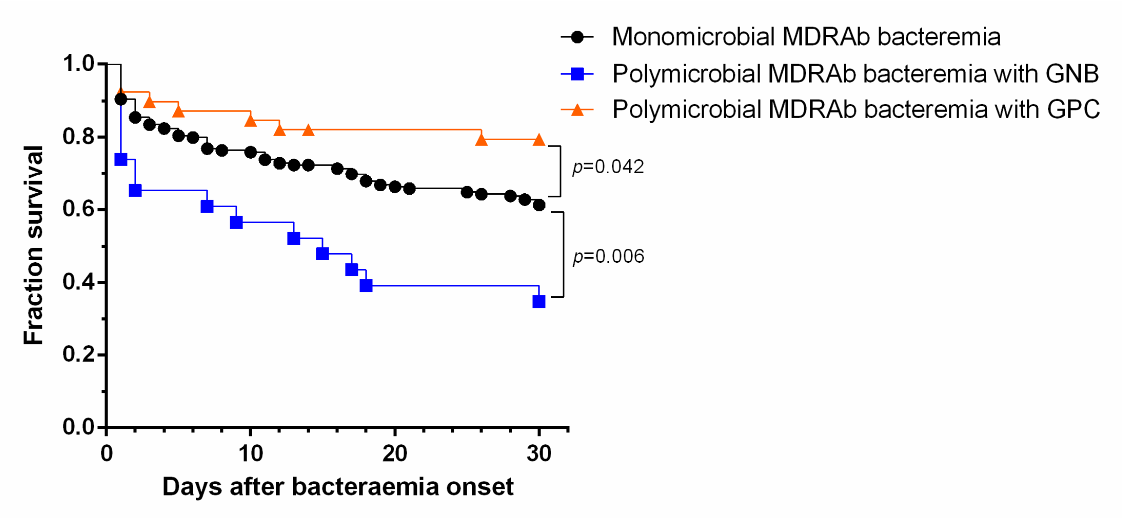

Supplement: Supplementary file 1 [file jcm-09-00153-s001.pdf]
